# Cells and Their Relation to Organisms

**Published:** 1883-02

**Authors:** Geo. A. Piersol

**Affiliations:** A Lecture delivered before the Longford Scientific Association.


### ARTICLE III.

### *Cells and Their Relation to Organisms.*

BY GEO. A. PIERSOL, M. D.,

A Lecture delivered before the Longford Scientific Association.

While the hope to discover the essential principle of life formed a never failing spring of inspiration and stimulus to ceaseless labor and speculation on the part of those ancient philosophers, whose acuteness and accuracy, even now excite our wonder and admiration, and while in the domains of mathematics, physics, and astronomy their labors were frequently crowned by the demonstration of thruths, which the elapse of ages have rendered but still more indisputable, yet to those never tiring workers the true minute structure of the tissues—the seat of those vital phenomena, whose explanation was so earnestly sought—remained as deep and unravelled a mystery as the Elixir Vitœ.

The earliest magnifying lens—if indeed it can be called such—is probably that exhumed from the palace of Nimrud; it is of rock-crystal, rudely shaped, and probably was never used for optical purposes. Aristophanes tells us that "burning sphere" were sold in the shops of Athens nearly 500 years before the Christian era, but, nothwithstanding a long acquaintance with lenses, and some of their properties, there is no evidence that they were employed for investigation; not until long after in the 17th century, when in 1656, Borrellus won for himself the distinction of being the pioneer histologist, by, probably, first applying the microscope to the revelation of the structure of animal tissues. To Leeuwenhoek, however, over a century later, must be applied the title of "Father of Histology," as he was the first who instituted a systematic examination of the various tissues aided by this instrument.

About this time numerous observers had entered the field of microscopical research, the accuracy of whose deliniations of objects, in some instances, to-day stand unimpeached, while the explanations and interpretations accompanying may be even absurd in the light of our present knowledge. While the descriptions of structures as seen under the instrument were rapidly accumulating it was not until about the middle of the 18th century, that we find the proposition advanced that the tissues are formed from ultimate elements, corresponding more or less with the ultimate particles of the inorganic world.

About 1760, Haller declared that all tissues were resolvable into primary units, and these he declared to be the "fibre" and "organized concrete," assigning, however, to the fibre the more important role. This "fibre" theory was quite generally accepted until the close of the century when a reaction by the advancement of the "globular" theory, gave the signal for a contest which waged with vigor for nearly sixty years, during which time the names of those then mos tfamous in the study of natural history were drawn into the mælstrom of contention.

It was, however, reserved for Schleiden and Schwann, about 1837, to announce these observations by which it was demonstrated that all tissues are built up from "cells" formed after an uniform methed; this was first pointed out by Schleiden regarding vegetable tissues, and almost immediately applied by Schwann to animal structures; the investigations of these observers thus lying the foundation for our modern cell doctrine.

Without pausing to discuss the interesting consideration regarding the individual parts of the cell, as presented by these authors, we may briefly sum up the typical cell as conceived by them as being a closed vesicle always enveloped by a distinct membrane, containing more or less fluid cell contents, in which almost invariably was found a smaller, eccentric round body, the nucleus, containing within itself a third minute body, the nucleous. Of the cell, the nucleus and the nucleous were considered to be essential parts, especially the nucleous from which the cell had its origin and began its growth, the nucleous and cell-contents being subsequently formed in their respective order.

Without considering the gradual evolution of our present views regarding the cell, in the modern conception of the physiological elementary unit, a cell may be defined in the language of Prof. Tyson, as a minute "mass of living matter possessing the essential life properties of reproduction, nutrition, growth and development."

The typical animal cell is represented by the ovum of the higher mammalia. Under the first microscope we have such a cell. Constituting it, we notice a cell-wall, or limiting membrane; cell-contents, in which is embedded a nucleus, in which further is seen the minute nucleous.

While this ovum presents all the parts of the typical cell, yet they, by no means, are always present, and, indeed, we shall see that in accordance with the definition adopted, all saw the cell-contents may be wanting, and still the structure is embraced in the term cell.

The cell-wall is generally absent: in many cells, however, there appears to be a peripheral hardening of the cell-contents, which thus forms a kind of envelope, denser than the ordinary cell-contents. The latter is usually a soft, semi-fluid albumenous substance, more or less clear and homogeneous and containing numerous fine particles or granules, which latter may be colored thereby giving a decided tinge to the cell, as is here seen in the so-called pigmental epithelium of the retina.

Regarding the "granular" appearances of the cell-contents, as well as of the nucleus, as usually described, it may be mentioned that during the last few years, largely through the labors of Heitzmann, Klein, and Flemming, as well as of many other observers, this "granular" appearance is claimed to be the expression under low powers of very delicate networks, which exist; the cross-sections and points of intersection of the fibres of which producing the well-known granules.

The nucleus, which is usually present, presents a more densely granular appearance, and possesses the property of taking a deeper coloration than the other parts of the cell on the addition of many coloring solutions, as those of carmine, logwood, and many of the aniline dyes. The so-called nucleolus is variable in its presence, being probably less frequently seen, that satisfactorily demonstrable, according to the views of Klein, the appearance represents no real separate part of the cell, but only a condensation or thickening of the delicate net-work, which, as just stated, by some observers is claimed to exist normally in all cells. especially in the nucleus. We have never been able to accept this explanation of the nucleolus. That in many cells this structure is absent, or at least but questionably present must be admitted, but, on the contrary, it is difficult to believe that the beautifully distinct nucleoli, as seen in nerve and epithelial cells, are but shrunken and aggregated fibrils; while the decided difference in color from that of the nucleus as seen in tissues doubly stained, and even in gold preparations strengthens the conviction of the reality of the nucleolus as a distinct part of the cell.

The different parts of the cell vary in regard to their relative vitality, and they, doubtless, are probably different in chemical composition. If we stain any typical cell with a solution of carmine not all parts of the cell take the dye with equal intensity; the nucleus is more deeply stained than the remaining parts of the cell—the cell-contents. This property of appropriating with avidity carmine and allied dyes is peculiar to those parts possessing the greatest vitality—the "living-matter," the "germinal-matter," or the "biophlasm." If we take a cell from the surface of a mucous membrane, as that lining the mouth, and stain it, we will find that but a relatively small part of the cell becomes deeply colored, that part being the nucleus, while the cell contents takes but a faint tinge of color. Such a cell is shown under the microscope. The nucleus is here alone the living matter or bioplasm of such a cell. Now age greatly modifies the amount of the living-matter present in the cell. In the case just cited of the epithelial covering of mucous membranes, the deeper layers represent the youngest cells, on examining cells from these, the nucleus, which represents the bioplasm, is found to be relatively much larger than in the old, mature cells: there we saw the living matter very small, here the nucleus occupies the greater part of the entire cell, and it is also to be noticed that even the cell-contents take a deeper tinge than in the old cells. Of this difference in the intensity with which the parts of the cell stain according to age, advantage is taken in determining the relative ages of cells—the farther removed from youth the less the amount of living-matter, and the less intensity of the color.

Now while cells, as a rule, are formed but in part of bioplasm, some cells are composed entirely of the living-matter, as the white blood corpuscle and the so-called indifferent or lymphoid cell: in these we have not only deep coloration on staining, but also actual manifestations of life in the changes in form and place, which may be observed readily under favorable conditions, and which, from their resemblance to those of that primative organism and amœba, are known as amœboid movements.

Reproduction, it will be remembered, was one of the vital phenomena attributed to cells in our definition, and in it we have presented the most important role of the unit. Schleiden and Schwann. while recognizing much of the life-history of the cell, believed they originated often spontaneously; Virchow, however, later announced the truth that every cell is derived from some previously existing cell, formulating the now classic phrase *omnis cellula e cellula*, derived from the older formula *omne vivum ex evo.*

Cells reproduce and multiply their kind by several modes.

1st. *By fission* or division; in the simplest case dividing and setting free a portion of themselves as new cells capable of all the functions of the parent, as often takes place in those cells which are almost wholly of bioplasm, as the white blood-cells: the more usual mode by fission, however involves the nucleus. The nucleus first elongates, becomes constricted, and finally separates into usually two—sometimes more—portions, which separation is subsequently followed by a corresponding cleaving of the body of the cell, thus eventually giving rise to two or more distinct cells instead of the original single one. Under the instrument his is well exhibited in the growing cartilage of a young frog.

2nd. *By gemmation*, or a process of budding, as is illustrated in the growth of the yeast plant under the next microscope;

3rd. *By endogenous multiplication*, where we have a new progency of cells formed within the parent cell without a division of the body of the original cell: this is shown under the instument. The last two modes of reproduction, however, are very much less common in animal tissues than the first.

During the last few years our knowledge of the method of the division of nuclei and cells has been largely extended through the elaborate studies of Strausburger and Flemming, and subsequently by many other observers. According to these researches the process of division is far from being the simple changes heretofore recognized, since the study of the changes taking place in the elements of the dividing nucleus—Karyokinesis—reveals a far greater complication that before supposed. These researches, by the way, are among the practical triumphs of recent objectives of the widest angles as rendered possible by the introduction of "homogeneous" immersion fluids; to the superb definition of such lenses, as made by Zeiss, Tolles, Powell and Leland, Seibert, and others, all bear testimony.

Having thus briefly considered the chief characteristics of the cell, let us discuss its relation to organisms and the modifications to which it is subjected in the course of higher development. It need not be said that to highly developed animals the individual cell bears but a very insignificant relation to the whole, but as we decend the scale of life we find the cell assuming a more and more commanding position, until in the lowest stage of life, the cell constitutes the entire organism. Under the microscope we have an amœba, which in its quiescent stage is but a single cell, generally round and mostly with a nucleus; in its more active form the vital phenomena are most marked, the organism improvising such special organs as may become necessary for the performance of its functions. A little higher and we find in the "sea-jellies," which abound in tropical waters, a number of cells necessary to the perfection of the individual; and as the animal scale is ascended, we find a greater and greater number of cells taking part until in the organism are untold millions, each contrbuting, its part to the formation of the perfect whole: in addition as we advance, division of labor takes place, producing modification of structure for specialized function—certain cells become modified to perform certain and exclusive duties.

In the history of the development of the higher animals, there is a period when no morphological difference exists between the cells composing the germinal mass; soon, however, certain layers are formed, which further and further develop until from a mass of cells undistinguishable in their similarity, we have cells produced as different in appearance and function as the large ganglionic cells of the spinal cord, and those of involuntary muscle. Under the succeeding microscope is a section of an early embryo chick, in which, while systems and organs are beginning to be mapped out with some differentiation of the cells, yet they all present a marked similarity.

As has been already intimated, the form of the typical free cell is round, this brings the usual form of the youngest cells: where, however, they become crowded together in their development they exert mutual pressure, and the curved surfaces of contact give place to straight ones, thus producing the polygonal figures so characteristic of some forms of the covering of mucous membranes—epithelium. Other cells are never subjected to pressure, and are developed according to their intended function into spindle, multipolar, and other forms.

The slides under the succeeding microscopes will serve as illustrations of the changes in form which cells undergo from being the embroyonal indifferent element.

First, we have those which may be called cells *of nutrition*, as represented by those of the blood and lymph: here but comparatively slight change has taken place; most markedly in the red corpuscles, scarcely at all in the white blood-cells, which are considered as identical with those of the lymph, and which are the normally present type of the embroyonal cells, which they closely resemble,

Secondly, *cells forming coverings.* 1st of the *integument*, 2nd, of the *mucous membranes*, including the squamous, columnar, and that curious modification of the latter, the ciliated variety. These latter by the constant motion of minute hair-line appendages create a current, by which the objects in contact are propelled in definite directions, a notable example of these being the lining of the respiratory tract, where expulsion of the accumulating mucous is necessary. In the lower forms of life these minute cilia form an efficient means of obtaining nourishment, by creating currents by which food is drawn to the animal. 3rd, of the *serous membranes* where the cells form large flat plates, usually distributed in a single layer, while the coverings of the mucous membranes consist of several. As modifications of the cells of mucous membranes those constituting glands, the so-called glandular epithelium, present themselves.

Thirdly, *cells of the connective-substances* whose forms vary from oval to beautifully stellate, according to age and development : of these beautiful examples are exhibited as found in embryonal tissues and in the cornea.

Fourthly, the import group of *cells of the muscular system* : in the voluntary muscle and in tendon are striking examples of departure by development from the original form of the cells : in the involuntary muscle cells, here taken from the mesentery of the newt, is a beautiful illustration of the spindle or lanceolate form.

Fifthly, *cells of the nervous system.* We here have some of the splendid branched nerve-cells formed in the spinal cord, each in communication with nerves, and with each other, and capable of originating within themselves nervous force ; next represented are the cells taking part in the formation of the cerebral mass, including those of the cerebrum, cerebellum, and pons varolii : in addition to these is a slide of typical gangloin cells of the sympathetic nervous system.

As interesting modifications, the cells forming the terminal organs of special sense present themselves, where function is again seen to deeply impress the cells: we have here those of touch and taste, smell and sight, as represented in the Pacinian corpuscle, taste-buds, Schneiderian membrane, and the retina.

In this hasty, and by no means complete, consideration of the cell, we have seen that the typical cell is composed of cell-wall, cell-contents, and nucleus, and sometimes nucleous; that in many instances one or more of these parts may be wanting, and yet the term "cell" may be applied: that usually the nucleus, sometimes, however, the entire cell, is the seat of vital manifestations—that the nucleus is composed of the living-matter or bioplasm; that while in the lower forms of life the cell may constitute the individual, the higher we ascend the scale the greater the specialization of function and a corresponding modification of those cells to which now are assigned special duties; and, finally, that, while at one stage in the existence of the most highly developed organization all the component cells were morphologically similar and undistinguishable, yet in the course of growth and development into the capability of performing their assigned roles, their forms undergo great and permanent alterations.—*Western Med. Reporter.*

---

